# ST2^+^ Erythroid Progenitors Suppress Allergic Asthma by Scavenging IL‐33 in Young Mice

**DOI:** 10.1002/clt2.70111

**Published:** 2025-10-17

**Authors:** Chang Li, Jie Liu, Xiaoshi Li, Wenlong Chen, Ying Zhang, Heng Sun, Cunni Zheng, Quan Liu

**Affiliations:** ^1^ Department of Biochemistry, Key University Laboratory of Metabolism and Health of Guangdong, Joint Laboratory of Guangdong‐Hong Kong Universities for Vascular Homeostasis and Diseases, SUSTech Homeostatic Medicine Institute School of Medicine Southern University of Science and Technology Shenzhen China; ^2^ Flow Cytometry Core Facility Shenzhen Medical Academy of Research and Translation Shenzhen China; ^3^ Department of Oncology National Cancer Center/National Clinical Research Center for Cancer/Cancer Hospital & Shenzhen Hospital Chinese Academy of Medical Sciences and Peking Union Medical College Shenzhen China

**Keywords:** early‐onset allergic asthma, IL‐33, neonatal mice, ST2^+^ erythroid progenitor (EP)

## Abstract

**Background:**

Although IL‐33/ST2 signaling has been implicated in adult asthma, its contribution to early‐onset allergic asthma remains poorly understood. Here, we examined the postnatal dynamics of IL‐33 and its regulation by splenic ST2^+^ erythroid progenitors (EPs).

**Methods:**

Plasma IL‐33 levels were measured in neonatal and young mice. Wild Type (WT) and *Il33* ^−/−^ mice were exposed to house dust mite (HDM) to assess airway inflammation and asthma. ST2^+^ EPs were analyzed for IL‐33 responsiveness, transcriptomic/epigenomic profiles, and IL‐33 scavenging capacity. EP depletion was performed to evaluate their role in HDM‐induced inflammation.

**Results:**

Plasma IL‐33 levels fluctuated during the postnatal period, peaking at postnatal day 7 (PND7). WT mice exhibited more severe HDM‐induced airway inflammation than *Il33* ^−/−^ mice. Splenic ST2^+^ EPs, abundant in early life but absent by PND28, displayed minimal IL‐33–induced signaling or transcriptomic/epigenomic alterations, yet efficiently scavenged IL‐33. Depletion of EPs exacerbated HDM‐induced inflammation, accompanied by increased T follicular helper cells (Tfh) and IgE^+^ B cells.

**Conclusion:**

ST2^+^ EPs function as transient IL‐33 scavengers during early life, attenuating its pro‐asthmatic effects and preserving immune homeostasis.

**Abbreviations**
AM: Alveolar macrophage
BM: Bone marrow
EP: Erythroid Progenitor
HDM: House Dust Mite
ILC2: Group 2 Innate Lymphoid Cell
MLN: Mediastinal Lymph Node
PND: Postnatal Day
sST2: Soluble ST2 Receptor
Tfh: T Follicular Helper Cell
WT: Wild Type

## 1 Background

Interleukin‐33 (IL‐33), a nuclear member of the IL‐1 cytokine family [1, 2], is constitutively expressed by structural cells such as epithelial cells, fibroblasts, and glial cells across multiple species [3, 4, 5]. It functions both as a chromatin‐associated nuclear factor and as an extracellular alarmin, thereby playing dual roles in tissue homeostasis and immune activation. IL‐33 signals through a receptor complex composed of ST2 and IL‐1RAcP [6, 7]. In asthma, epithelial‐derived IL‐33 promotes type 2 inflammation via direct activation of ST2^+^ immune cells—such as group two innate lymphoid cells (ILC2s) and Th2 cells—enhancing eosinophil recruitment and mucus hypersecretion [8]. Clinical trials have demonstrated that IL‐33 neutralization improves outcomes in moderate‐to‐severe asthma [9, 10].

Allergic asthma commonly develops during early life, a critical window for immune and lung development. Genome‐wide association studies have consistently identified IL33/IL1RL1 as major genetic determinants of early‐life–onset asthma susceptibility [11]. Although adult murine models have elucidated the pro‐inflammatory roles of IL‐33, key questions remain regarding its regulation during postnatal immune maturation. Notably, asthma phenotypes often persist in children even after allergen withdrawal, suggesting distinct developmental programming compared to adults. According to epidemiological data, the prevalence of asthma symptoms in children and adolescents is approximately 10%, while in adults it ranges from 6% to 7%. There are significant variations across countries, with the prevalence in adults ranging from 2% to 3% in low‐income countries to 10% in high‐income countries [12]. Moreover, the proportion of allergic asthma cases declines progressively with age [13].

Our study bridges this knowledge gap by identifying a developmentally restricted population of ST2^+^ erythroid progenitors (ST2^+^EPs) that emerge during postnatal weeks 1–4 in murine spleen and bone marrow (BM). Although these cells express ST2, they exhibit minimal transcriptional response to IL‐33 and instead function as potent IL‐33 scavengers in vivo. We propose that ST2^+^ EPs act as physiological scavengers of IL‐33 during early immune development, mitigating its pro‐inflammatory effects and protecting against HDM‐induced allergic asthma. Our findings reveal a novel mechanism through which early erythropoiesis contributes to immune homeostasis during postnatal development.

## 2 Methods

### 2.1 Animals

*Il33* ^−/−^, *Il1rl1* ^−/−^ male and female C57/6N mice were obtained from the Jackson Laboratory and maintained under specific pathogen‐free conditions. All animal procedures were conducted in accordance with institutional ethical guidelines approved by the Laboratory Animal Center of Southern University of Science and Technology. Pregnant females were monitored twice daily to determine the timing of delivery.

### 2.2 Tissue Preparation and Cell Isolation

Spleens and lungs were harvested, mechanically dissociated in cell staining buffer (CSB) containing 0.3% BSA, and filtered through 70 μm cell strainers. After centrifugation, red blood cells (RBCs) were lysed where appropriate, and single‐cell suspensions were prepared for flow cytometry. RBC lysis is not required for EPs.

BM was flushed from femurs and tibias using CSB with 0.3% BSA and filtered similarly. Cell suspensions were centrifuged, RBCs lysed as needed, and resuspended in CSB for subsequent staining procedures.

### 2.3 Flow Cytometry

Cells (2‐5 × 10^6^) were incubated with Zombie NIR dye (BioLegend, 423106) in CSB with 0.3% BSA for viability staining, followed by Fc receptor blocking using anti‐CD16/32 antibodies. After 10 min at 4°C, fluorophore‐conjugated antibodies were added for 30 min in the dark. Cells were washed and resuspended in PBS + 0.3% BSA. Data were acquired using a flow cytometer and analyzed with FlowJo software. All antibodies were purchased from BioLegend unless otherwise indicated (Supporting Information S1: Table S1).

### 2.4 Phosphoflow Cytometry

Single‐cell splenocyte suspensions were prepared in RPMI supplemented with 10% FBS and filtered through 40 μm strainers. After cell counting, 1 × 10^6^ cells were resuspended in 400 μL culture medium and incubated at 37°C with 5% CO_2_. Cells were stimulated with PMA and ionomycin at various time points (0, 20, 30 min), fixed in 8% paraformaldehyde for 10 min at 37°C, and chilled on ice. After washing, surface staining was performed in 100 μL staining buffer (1 h, room temperature, dark), followed by permeabilization in 90% methanol at 4°C. Intracellular staining was conducted using phospho‐specific antibodies (NF‐κB p65, clone 93H1, Cell signaling, 4887s; JNK, clone G9, Cell signaling, 9257s; ERK1/2, clone 197G2, Cell signaling, 13148s; p38, clone 28B10, Cell signaling, 4552S). Cells were washed and resuspended in PBS prior to analysis (Supporting Information S1: Table S1 and S2).

### 2.5 ELISA

Plasma IL‐33 concentrations were quantified using the Mouse IL‐33 Uncoated ELISA Kit (Thermo, 88‐7333) according to the manufacturer's protocol (Supporting Information S1: Table S2).

### 2.6 RNA Sequencing

Splenic EPs were sorted from postnatal day 3 (PND3) and PND7 Wild Type (WT) and *Il1rl1* ^−/−^ mice using FACS. Cells were pelleted, lysed in TRIzol reagent (Thermo Fisher), and processed for RNA extraction and sequencing following standard protocols (Supporting Information S1: Table S2).

### 2.7 Tissue Fixation and Freezing

Mouse spleens were collected and lungs were perfused with PBS followed by 2% PFA via the inferior vena cava. Tissues were fixed in 2% PFA at 4°C for 2 h, then dehydration in 30% sucrose (4°C, 24 h) with solution changes every 6 h. Finally, tissues were embedded in OCT for frozen section preparation (Supporting Information S1: Table S2).

### 2.8 Immunofluorescence

Frozen sections (6 μm) were rehydrated, fixed in 2% PFA, permeabilized in 0.1% Triton X‐100, and blocked with 20% donkey serum. Sections were incubated with primary antibody (1:200, anti–IL‐33, R&D, AF3626; Ter119, clone TER‐119, BioLegend, 116215; ST2, clone U29‐93, BD, 566309), followed by secondary fluorophore‐conjugated antibodies (1:200, BioLegend, A32758). After DAPI (R&D, 2507021) staining, sections were mounted in antifade medium and imaged using a Nikon A1R confocal microscope (Supporting Information S1: Table S1).

### 2.9 Young Asthma Model

On PND3, neonatal mice were intranasally sensitized with 1 μg house dust mite (HDM) (Greer Labs) in 5 μL PBS, followed by five daily challenges of 10 μg HDM in 10 μL PBS. Mice were sacrificed within 3 days of the final challenge. Lungs were lavaged and perfused via the right ventricle with PBS, then processed for flow cytometry.

Antibody‐mediated EP depletion:

To deplete CD71^+^ EPs, mice received escalating doses of anti‐CD71 antibody or control rat IgG (Supporting Information S1: Table S1): 20 μg (PND2), 40 μg (PND4), 80 μg (PND6), and 160 μg (PND8). Tissues were harvested at PND10 and PND18 for analysis.

## 3 Results

### 3.1 Age‐Dependent Fluctuations in IL‐33 Levels Drive Type 2 Immune Responses During Early‐Life Sensitization in Mice

**FIGURE 1:**
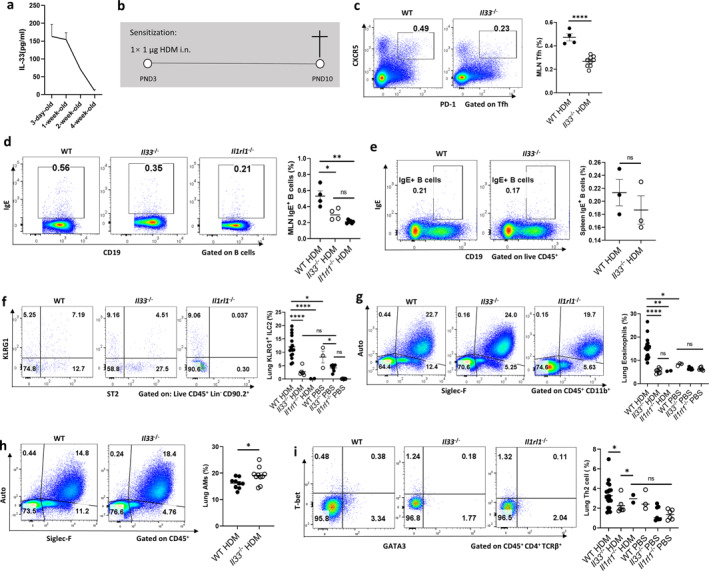
IL‐33 dynamics and its role in HDM‐induced asthma sensitization in young mice. (a) Plasma IL‐33 concentrations in WT mice at different ages (ELISA). (b) Schematic of HDM‐induced asthma sensitization model in young mice. Mice were sensitized with intranasal HDM at PND3, and detection was performed at PND10. Flow cytometry analysis of WT and Il33^−/−^ mice after HDM or PBS sensitization: (c) Tfh cell proportion (MLN; Zombie^−^ CD45^+^ CD4^+^ TCRβ^+^ PD‐1^+^ CXCR5^+^). (d, e) IgE^+^ B cell proportion in MLN (d) and spleen (e) (Zombie^−^ CD45^+^ CD19^+^ IgE^+^). (f) KLRG1^+^ ILC2s (lung; Zombie^−^ Lin^−^ CD45^+^ CD90.2^+^ ST2^+^ KLRG1^+^). (g) Eosinophils (lung; Zombie^−^ CD45^+^ CD11b^+^ Auto^−^ Siglec‐F^+^). (h) Alveolar macrophages (lung; Zombie^−^ CD45^+^ Auto^−^ Siglec‐F^+^). (i) Th2 cells (lung; Zombie^−^ CD45^+^ CD4^+^ TCRβ^+^ GATA3^+^). Data are mean ± SEM; ns, *p* > 0.05; **p* < 0.05, **p* < 0.01, ***p* < 0.001, ****p* < 0.0001.

We observed dynamic fluctuations in IL‐33 levels in murine plasma across developmental stages. To characterize this temporal pattern, we collected plasma from WT mice at postnatal days 3, 7, 14, and 28 and measured IL‐33 concentrations using ELISA. The results demonstrated that WT mice exhibited an early postnatal IL‐33 surge, with peak concentrations occurring within the first week of life, followed by a progressive age‐dependent decline to near‐undetectable levels in 4‐week‐old mice (Figure 1a). Consistent with previous reports of IL‐33 surges in neonatal lungs, we observed elevated plasma IL‐33 levels peaking in the first week of life. [14, 15], potentially due to mechanical stress from lung inflation and programmed neonatal cell death releasing IL‐33 as an alarmin. This postnatal spike occurs during a critical window of immune immaturity, raising questions about its impact on immune development and asthma susceptibility.

To investigate IL‐33's role in early‐onset asthma mechanism, we established an HDM‐induced allergic sensitization model (Figure 1b) and compared WT and *Il33* ^−/−^ mice. At post natal day 10 (PND10), WT mice exhibited significantly higher proportions of lung dendritic cells (DCs) and activated CD86^+^ DCs (Supporting Information S1: Figure S1a–c), indicating enhanced antigen presentation [16, 17]. T follicular helper (Tfh) cells were increased in WT mediastinal lymph node (MLN) (Figure 1c), correlating with elevated IgE^+^ B cell frequencies (Figure 1d), a hallmark of allergic sensitization. [18, 19, 20, 21, 22]. In contrast, *Il33* ^−/−^ and *Il1rl1* ^−/−^ mice showed diminished Tfh and IgE^+^ B cell responses.

After sensitization at PND3, WT mice displayed robust increases in IgE^+^ B cells across the MLN, lungs, spleen, and BM (Figure 1d,e, Supporting Information S1: Figure S1d,e). *Il33* ^−/−^ mice exhibited reduced IgE^+^ B cells in most tissues, except the spleen, indicating tissue‐specific IL‐33 signaling.

Besides, ILC2s, major responders to IL‐33, are recruited to neonatal lungs where they produce IL‐4, IL‐5, and IL‐13 [23]. We observed significantly increased KLRG1^+^ ILC2s in WT lungs following HDM exposure, which were reduced in *Il33* ^*−/−*^ mice (Figure 1f). This immune activation led to increased eosinophil recruitment (Figure 1g), impaired alveolar macrophage (AM) function (Figure 1h), and elevated lung Th2 cells in WT mice, all of which were significantly attenuated in *Il33* ^−/−^ or *Il1rl1* ^−/−^ mice (Figure 1i). Collectively, these data establish IL‐33 as a central driver of early‐life type 2 immunity and allergic sensitization.

### 3.2 IL‐33 Drives Type 2 Immunity in Young Mice Allergic Asthma

**FIGURE 2:**
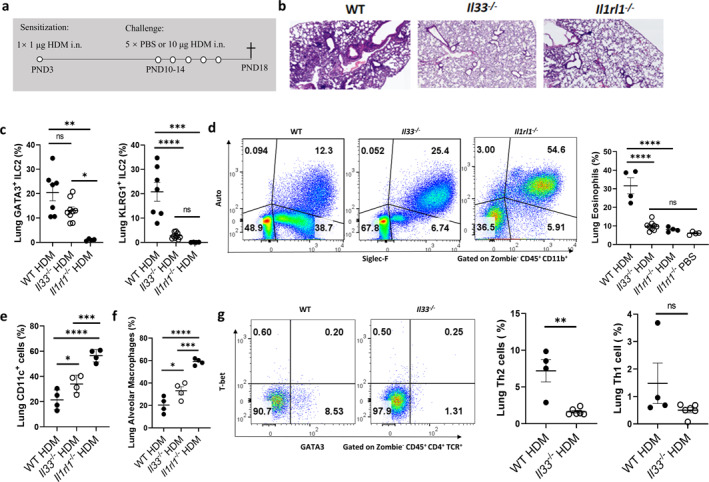
IL‐33/ST2 axis regulates asthmatic pathology and immune responses in young mice. (a) HDM‐induced asthma model in young mice. (b) Lung H&E staining. (c) GATA3^+^ ILC2 (Zombie^−^ Lin^−^ CD45^+^ CD90.2^+^ ST2^+^ GATA3^+^) and KLRG1^+^ ILC2 (Zombie^−^ Lin^−^ CD45^+^ CD90.2^+^ ST2^+^ KLRG1^+^). (d) Eosinophil (Zombie^−^ CD45^+^ CD11b^+^ Auto^−^ Siglec‐F^+^). (e) CD11c^+^ cell (Zombie^−^ CD11c^+^). (f) AMs (Zombie^−^ CD45^+^ CD11b^lo/–^ CD11c^+^ Siglec‐F^+^ Auto^+^). (g) Th2 (Zombie^−^ CD45^+^ TCRβ^+^ CD4^+^ GATA3^+^) and Th1 (Zombie^−^ CD45^+^ TCRβ^+^ CD4^+^ T‐bet^+^) cells. Data are mean ± SEM; ns, *p* > 0.05, *p* < 0.05, **p* < 0.01, ***p* < 0.001, ****p* < 0.0001.

To further elucidate the functional role of IL‐33 in early‐life asthma, we established a HDM‐induced allergic asthma model (Figure 2b), previously reported to induce severe bronchial and pulmonary vascular inflammation as well as elevated serum HDM‐specific IgG [14] (Figure 2a, Supporting Information S1: Figure S1i,j). Histopathological examination revealed significantly more severe asthma phenotypes in WT mice compared to *Il33* ^−/−^ counterparts, as evidenced by bronchial wall thickening, airway constriction, and dense peribronchial and perivascular inflammatory infiltrates (Figure 2b). These features are consistent with classical eosinophilic asthma.

Mechanistically, HDM exposure resulted in similar increases in total ILC2 numbers in both genotypes, but activation of KLRG1^+^ ILC2s was markedly diminished in *Il33* ^−/−^ mice (Figure 2c), indicating IL‐33's specific role in promoting ILC2 effector function. Moreover, lung eosinophil recruitment was significantly reduced in *Il33* ^−/−^ mice, with eosinophil levels approaching those seen in PBS controls (Figure 2d), underscoring IL‐33's centrality in type 2 inflammation. We also observed immune regulatory effects on the AM population. HDM‐challenged WT mice exhibited substantial reductions in CD11c^+^ AMs (Figure 2e) and showed signs of AM dysfunction (Figure 2f). In contrast, *Il33* ^−/−^ and *Il1rl1* ^−/−^ mice retained higher numbers of functional AMs, suggesting that IL‐33 impairs macrophage in the inflamed lung microenvironment. The type 2 immune bias was further corroborated by increased frequencies of GATA3^+^ Th2 cells in the lungs of WT mice, without compensatory increases in Th1 populations (Figure 2g). This indicates a selective skewing toward type 2 responses driven by IL‐33, likely through ST2‐mediated activation of effector Th2 cells.

Interestingly, intranasally administered allergens‐OVA 647 were found to distribute systemically, particularly accumulating in the spleen (Supporting Information S1: Figure S2a–d). This suggested that neonatal mice mount systemic immune responses to environmental allergens, with splenic and BM immune cells exhibiting immunological engagement.

Taken together, these findings demonstrate that IL‐33 orchestrates early allergic inflammation by simultaneously activating innate and adaptive type 2 immune pathways.

### 3.3 ST2^+^ EPs can Scavenge IL‐33 in Vitro in Young Mice

Despite the key role of IL‐33 in early‐onset asthma, the abundance of canonical IL‐33‐responsive cells such as ILC2s and mast cells remains relatively low in early life. Strikingly, we identified abundant CD71^+^ST2^+^ nucleated EPs in the spleen and BM of neonatal and young mice. These cells, not widely recognized in immunological contexts, displayed a developmental pattern that closely mirrored plasma IL‐33 levels. Both peaked during the first week of life and declined thereafter. This spatiotemporal correlation raised the possibility that ST2^+^ EPs might regulate local IL‐33 availability and influence asthma susceptibility.

**FIGURE 3:**
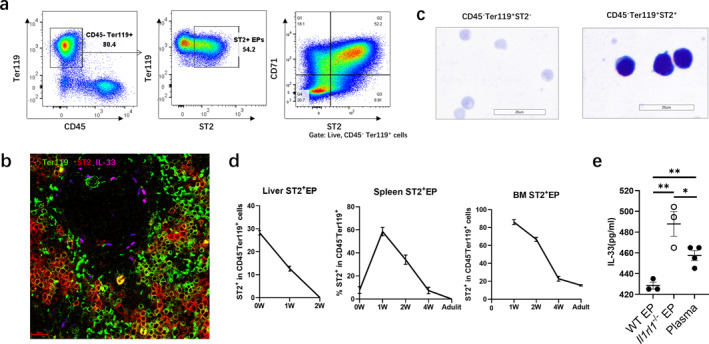
The spatiotemporal expression of ST2^+^ EPs in young mice. (a) Surface markers of ST2^+^ EPs in spleens of PND7 mice. (b) Immunofluorescence plots of ST2^+^ EP and IL‐33 in the spleens of young mice. Scale bars, 20 μm. (c) Giemsa staining of young mouse spleens for ST2^−^ EPs and ST2^+^ EPs. Scale bar, 20 μm. (d) Spatiotemporal distribution of ST2^+^ EPs in the liver (Lv.) and spleen (Sp.) of mice of different ages, proportions of ST2^+^ EPs in BM. (e) Splenic CD71^+^ EP of PND 7 WT or *Il1rl1* ^−/−^ mice were sorted and cultured with young plasma for 6 h, then IL‐33 concentration in plasma was detected by ELISA. Data are mean ± SEM; ns, *p* > 0.05, *p* < 0.05, **p* < 0.01.

Flow cytometric analysis showed that over 50% of splenic CD45^‐^Ter119^+^CD71^+^ EPs at PND7 expressed ST2 and retained nuclear structures, distinguishing them from ST2^−^ EPs, which lacked nuclei (Figure 3a–c). Spatiotemporal mapping revealed that ST2^+^ EPs were present in the liver, spleen, and BM at birth. Their numbers increased in spleen and BM, peaking at PND7, and gradually declined, with liver‐resident EPs becoming undetectable by PND14. By adulthood, splenic ST2^+^ EPs were absent, whereas BM populations persisted (Figure 3d). Notably, the abundance of ST2^+^ EPs in the spleen and BM positively correlated with plasma IL‐33 concentrations, supporting their potential regulatory role.

To assess whether ST2 expression confers IL‐33‐binding capacity, we co‐cultured splenic CD71^+^ EPs from WT or *Il1rl1* ^−/−^ mice with plasma from neonates. After 6 h, IL‐33 levels were significantly reduced in cultures containing WT but not *Il1rl1* ^−/−^ EPs, confirming ST2‐dependent IL‐33 clearance (Figure 3e). These findings support a model in which ST2^+^ EPs function as an IL‐33 sink in early life, potentially modulating allergic sensitization.

### 3.4 IL‐33 Exhibits Minimal Impact on the Differentiation and Function of ST2^+^ EPs in Young Mice

**FIGURE 4:**
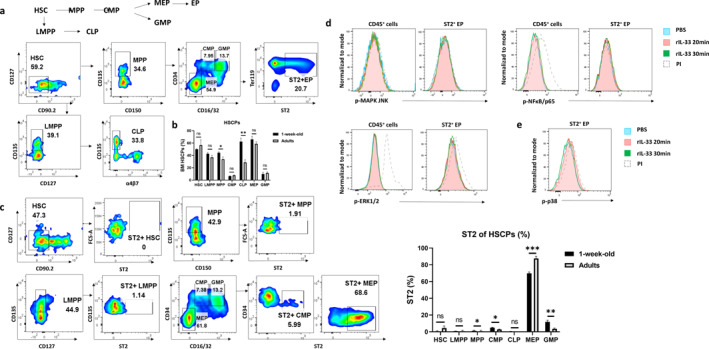
IL‐33 does not affect EP differentiation and function. (a) Schematic of murine hematopoietic differentiation pathway and representative flow cytometry plots of BM hematopoietic stem cells in adult mice. (b) Quantitative analysis of hematopoietic stem cell populations in BM of WT mice at 1 week postnatal and adulthood. (c) Flow cytometry analysis (left) and quantification (right) of ST2 expression across hematopoietic stem cell populations in BM of 1‐week‐old WT mice. (d) Intracellular p38 phosphorylation levels in splenic ST2^+^ EPs and CD45^+^ cells from 1‐week‐old WT mice following stimulation with 20 ng/mL recombinant IL‐33 (rIL‐33) for 20 or 30 min. (e) Time‐course analysis of p38 phosphorylation specifically in ST2^+^ EPs from 1‐week‐old WT mice spleen after rIL‐33 stimulation (20 ng/mL). Data are mean ± SEM; ns, *p* > 0.05, *p < 0.05, **p < 0.01, ***p < 0.001.

To explore the developmental origin and responsiveness of ST2^+^ EPs in neonatal spleens, we compared hematopoietic progenitor differentiation between PND7 and adult mice. Based on classical hematopoietic hierarchy, hematopoietic stem cells (HSCs) give rise to multipotent progenitors (MPPs), which further differentiate into common myeloid progenitors (CMPs) and common lymphoid progenitors (CLPs). CMPs subsequently generate granulocyte‐monocyte progenitors (GMPs) and megakaryocyte‐erythroid progenitors (MEPs) (Figure 4a).

Flow cytometry revealed substantial hematopoietic progenitor populations in both spleen and BM of PND7 mice, with higher frequencies in BM. As development progressed, progenitor frequencies, including MPPs and CLPs, declined in both tissues (Figure 4b). Notably, ST2 was expressed in several myeloid progenitors, including CMPs, GMPs, and MEPs, with > 60% of MEPs in neonatal BM and > 85% in adult BM expressing ST2 (Figure 4c). This enrichment supports the idea that ST2 expression is maintained during early erythroid commitment.

To determine whether IL‐33 activates downstream signaling in ST2^+^ EPs, we performed phospho‐flow cytometry on splenocytes from PND7 mice following recombinant IL‐33 (rIL‐33) stimulation. Among multiple conditions tested, only 20‐min exposure to 20 ng/mL rIL‐33 modestly increased p38 phosphorylation in ST2^+^ EPs (Figure 4e). Other signaling mediators, including JNK, ERK1/2, and NF‐κB, were not activated (Figure 4d). These results indicate that IL‐33 has minimal capacity to trigger canonical signaling cascades in ST2^+^ EPs under physiological conditions.

Together, these findings suggest that while ST2^+^ EPs arise from conventional erythroid lineages and express IL‐33 receptor components, they are functionally unresponsive to IL‐33 signaling in early life. Their primary role likely centers on passive IL‐33 clearance rather than dynamic signal transduction or immune regulation.

### 3.5 IL‐33 Exerts Minimal Epigenomic and Transcriptomic Effects on Splenic ST2^+^ EPs in Young Mice

To further elucidate whether IL‐33 influences the functional programming of ST2^+^ EPs, we performed integrated epigenomic and transcriptomic profiling of splenic ST2^+^ EPs isolated from PND7 WT and *Il33* ^−/−^ mice using ATAC‐seq and RNA‐seq.

Chromatin accessibility analysis revealed a broadly open epigenomic landscape at genes related to proliferation and immunoregulation, including *Cd24a*, *Cd47*, *Lgals9*, *Mki67*, *Tgfb1*. Conversely, loci associated with pro‐inflammatory mediators such as *Il1b*, *Ifng*, *Il6*, *Tnf* exhibited minimal accessibility (Extended Data Figure 3a), suggesting that ST2^+^ EPs are epigenetically poised toward a non‐inflammatory phenotype. Comparative analysis between WT and *Il33* ^−/−^ ST2^+^ EPs demonstrated no significant differences in global chromatin accessibility, indicating that IL‐33 is dispensable for the maintenance of their epigenomic state during early development.

Transcriptomic profiling similarly revealed negligible changes in gene expression between genotypes. Although *Il33* ^−/−^ ST2^+^ EPs showed modest upregulation of several metabolic genes (e.g., *Asns*, *Slc7a5*, *Shmt2*) and stress response regulators (e.g., *Dusp8*, *Atf5*) (Supporting Information S1: Figure S3b,c), enrichment analysis of differentially expressed genes did not yield statistically significant alterations in biological processes, cellular compartments, or molecular functions (Supporting Information S1: Figure S3e,f).

These data collectively demonstrate that IL‐33 signaling has little to no impact on the epigenetic or transcriptional programming of splenic ST2^+^ EPs in neonatal mice. Rather than being dynamic responders to IL‐33, these cells likely serve as a passive regulatory reservoir that modulates IL‐33 bioavailability through receptor‐mediated scavenging.

### 3.6 ST2^+^ EP Depletion Exacerbates HDM‐Induced Airway Inflammation During Sensitization and Asthmatic Phases in Young Mice

**FIGURE 5:**
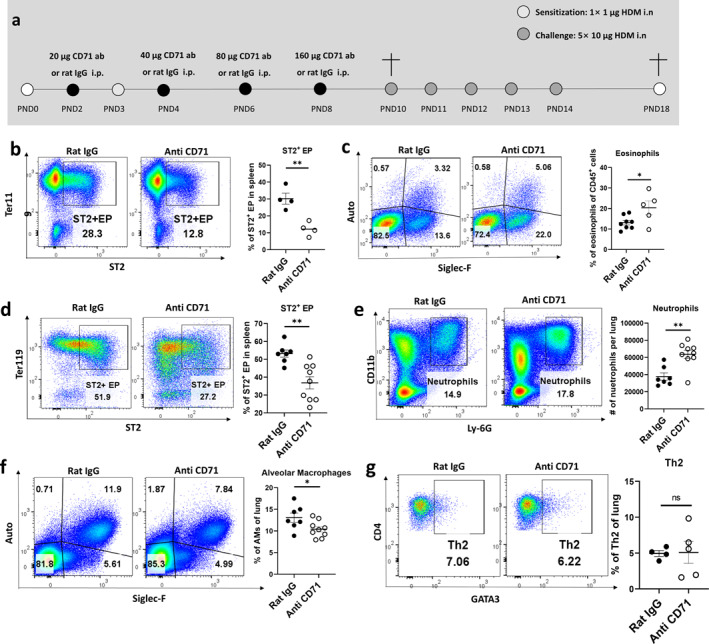
EP depletion alters immune responses during HDM sensitization and asthma in young mice. (a) Experimental scheme for EP depletion in HDM‐induced sensitization/asthma models. (b, c) Asthma model (PND18): splenic ST2^+^ EP (Zombie^−^ CD45^−^ Ter119^−^ ST2^+^) proportion (b) and lung eosinophils (Zombie^−^ CD45^+^ CD11b^+^ Auto^−^ Siglec‐F^+^) (c). (d–g) Sensitization model (PND10): splenic ST2^+^ EP proportion (d), lung neutrophils (Zombie^−^ CD45^+^ CD11b^+^ Ly‐6G^+^) (e), alveolar macrophages (Zombie^−^ CD45^+^ CD11b^lo/–^ CD11c^+^ Siglec‐F^+^ Auto^+^) (f), and lung Th2 cells (Zombie^−^ CD45^+^ CD4^+^ TCRβ^+^ GATA3^+^) (g). Data are mean ± SEM; ns *p* > 0.05, *p* < 0.05, **p* < 0.01.

Having established the IL‐33‐scavenging function of ST2^+^ EPs, we next evaluated their in vivo relevance using EP depletion models. We employed an anti‐CD71 antibody‐based strategy to selectively eliminate CD71^+^ EPs in neonatal mice during HDM sensitization and challenge (Figure 5a).

Flow cytometry confirmed effective depletion of splenic ST2^+^ EPs in antibody‐treated mice by postnatal day 18 (PND18) (Figure 5b). In the HDM‐induced asthma model, EP‐depleted mice exhibited significantly heightened airway inflammation, as indicated by increased eosinophilic infiltration in the lungs (Figure 5c). These results suggest that ST2^+^ EPs play a protective role by limiting IL‐33‐driven type 2 responses.

To dissect the underlying immune alterations during the sensitization phase, we analyzed immune cell populations at PND10 following EP depletion. As expected, ST2^+^ EPs in the spleen were markedly reduced (Figure 5d). EP‐depleted mice exhibited elevated pulmonary neutrophil levels (Figure 5e), indicative of intensified inflammation, and a concurrent reduction in AMs (Figure 5f), suggesting impaired tissue homeostasis. Interestingly, the frequency of Th2 cells in the lung remained unchanged at this early stage (Figure 5g).

### 3.7 Immunological Mechanisms Underlying the Exacerbation of Asthma in Young Mice After ST2^+^ EP Depletion

**FIGURE 6:**
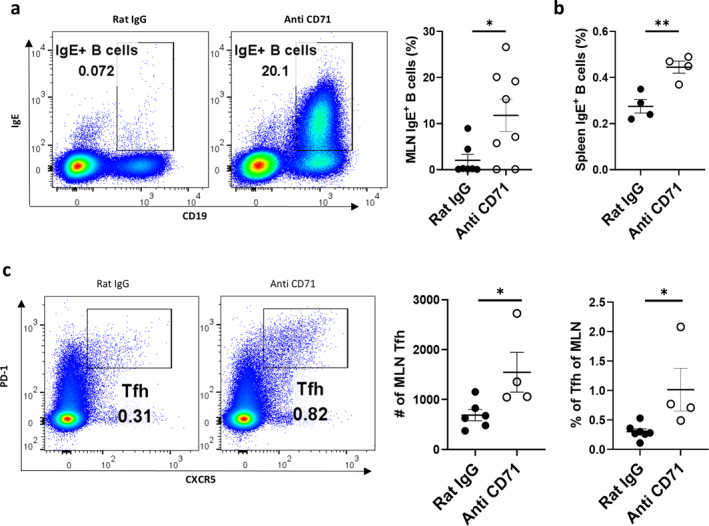
EP depletion enhances allergic sensitization in young mice. (a, b) Flow cytometry analysis of IgE^+^ B cells in MLN (Zombie^−^ CD45^+^ CD19^+^ IgE^+^) and spleen. (c) Tfh cells in MLN (Zombie^−^ CD45^+^ TCRβ^+^ CD4^+^ PD‐1^+^ CXCR5^+^). Data are mean ± SEM; *p* < 0.05, **p* < 0.01.

To investigate the immunological mechanisms by which ST2^+^ EP depletion exacerbates asthma pathogenesis in early life, we conducted detailed analysis of MLN immune cell dynamics during the sensitization phase. Our results demonstrate that anti‐CD71 antibody‐mediated ST2^+^ EP depletion significantly elevated IgE^+^ B cell populations in both MLN (Figure 6a) and spleen (Figure 6b). This finding presents a striking contrast to our previous observations showing comparable IgE^+^ B cell frequencies between WT and *Il33* ^−/−^ mice (Figure 1e), suggesting that splenic ST2^+^ EP may regulate IgE production through an IL‐33‐dependent mechanism.

Further characterization revealed that EP depletion markedly increased both the absolute number and relative proportion of Tfh in MLN (Figure 6c). Although we cannot be certain that Tfh cells are directly regulated by IL‐33, these data support a model in which ST2^+^ EP cells normally restrain allergic sensitization by limiting IL‐33 availability, and their depletion consequently enhances Tfh‐mediated B cell class switching, leading to amplified IgE production and exacerbated inflammatory responses. This regulatory pathway represents a novel mechanism by which EP can modulate adaptive immune responses in early life asthma development.

## 4 Discussion

Respiratory diseases remain the leading causes of infant mortality. Despite increased therapeutic efforts, the treatment of “Difficult‐to‐Treat Asthma” remains unsatisfactory, accounting for 50% asthma healthcare resources [24, 25]. The immune systems of children and adults [26], as well as the pathophysiology of asthma, differ, indicating that clinical trials for adults cannot be directly applied to children [27, 28, 29, 30]. Understanding the immune system of children is crucial to identify molecular targets for effective treatment.

In this study, we observed that ST2^+^ EPs were present in large numbers in the spleen and BM of PND7−14 mice. These cells gradually decrease in the spleen of adult mice and are eventually found only in the BM. We also confirmed that these cells differentiated from CMPs. We further demonstrated that IL‐33 promotes asthma in neonates via IgE production by B cells and increasing Tfh cell count. ST2^+^ EPs counteracted some pro‐asthmatic effects of IL‐33. This study provides the first evidence that ST2^+^ EPs play a protective role in early‐life asthma by scavenging IL‐33, thereby limiting eosinophilic inflammation and attenuating allergic airway disease. These findings not only advance our understanding of early‐life asthma pathogenesis but also identify the IL‐33/ST2^+^ EP axis as a promising therapeutic target, offering new avenues for developing immunomodulatory strategies against early‐life allergic asthma.

While defining markers for ST2^+^ EPs, we identified them as CD45^−^ immature erythroid progenitors. Flow cytometry revealed CD45^+^Ter119^+^ cell populations in the spleen and BM of young mice, previously linked to tumor immunosuppression. [31, 32, 33]. Confocal imaging of sorted CD45^+^Ter119^+^ cells showed they were heterodimeric aggregates rather than a distinct cell type (Supporting Information S1: Figure 1f). Although the role of ST2 in EPs remains underexplored, studies on CD71^+^ erythroid cells (CEC) highlight their immunosuppressive functions including promoting gut colonization, inhibiting CD11c^+^ cells, inducing Treg‐derived TGF‐β, modulating metabolism [34], and suppressing T cell proliferation through reactive oxygen species [34, 35, 36, 37, 38, 39, 40, 41, 42, 43, 44, 45, 46, 47]. Interestingly, ST2^+^ EP like cells have also been observed in the spleens of tumor‐bearing mice and patients [31, 48, 49], but unlike neonatal EPs, they lack immunosuppressive activity [50]. We did not investigate other ST2‐expressing immune cells in this study, such as regulatory T cells (Tregs) (Supporting Information S1: Figure 1h) because their abundance is much lower than that of EPs during this developmental stage. Although approximately 20% of BM‐derived CD71^+^ST2^+^ cells persist with age, we focused on the early postnatal period rather than the stage beyond PND18 for three reasons: first, EP numbers decline sharply after PND7, far below their peak abundance; second, the mouse immune system transitions to an adult‐like state after PND18, creating an inflammatory microenvironment distinct from that of early life; and third, the post‐PND18 period is characterized by a marked increase in immune cell abundance and very low plasma IL‐33 concentrations, reducing the necessity to demonstrate a role for EPs in IL‐33 regulation during this stage.

Regarding the role of soluble ST2 receptor (sST2), we acknowledge its potential protective function in regulating IL‐33. Previous studies have shown that sST2 levels are decreased while IL‐33 levels are elevated in asthmatic patients [51], Recombinant sST2 (r‐sST2) treatment administered to PND3 mice resulted in a reduction of serum total IgE and HDM‐specific IgG1 levels [14]. Our finding that ST2^+^ EPs protect against asthma by scavenging IL‐33 is not contradictory to this mechanism; rather, sST2 and ST2^+^ EPs may act synergistically, providing complementary modes of “cellular scavenging” and “soluble neutralization” that together maintain IL‐33 homeostasis during early life.

This study has several limitations. First, our findings are based on a neonatal mouse model, while species‐specific differences in immune cell composition, cytokine kinetics, and airway physiology may limit direct clinical translation. Second, we focused primarily on ST2^+^ EPs, whereas other ST2‐expressing immune populations, such as Tregs or ILC2s, were not systematically evaluated in this study. Third, we did not include longitudinal human samples, leaving the relevance of ST2^+^ EP dynamics and IL‐33 scavenging in infants unresolved. Future studies integrating human neonatal cohorts and functional assays will be necessary to validate the clinical significance of our findings and to explore potential therapeutic applications targeting the IL‐33/ST2^+^ EP axis.

## 5 Conclusions

Our study identifies splenic ST2^+^ EPs as transient but critical regulators of early‐life immune homeostasis. These cells, which peak in abundance during the first week of life, act as efficient IL‐33 scavengers and thereby restrain type 2 immune responses, limiting eosinophilic inflammation and allergic airway disease. Depletion of ST2^+^ EPs results in exacerbated HDM‐induced asthma phenotypes, accompanied by increased Tfh cell frequencies and IgE^+^ B cell responses. Together, these findings reveal a novel developmental mechanism linking erythropoiesis to asthma susceptibility and highlight the IL‐33/ST2^+^ EP axis as a potential therapeutic target for preventing or mitigating early‐onset allergic asthma during early life.

## Author Contributions

Chang Li contributed to manuscript revision and editing, drafting the initial version, experimental design, data collation, and visualization. Jie Liu participated in manuscript revision and technical support. Xiaoshi Li, Wenlong Chen, Ying Zhang, Heng Sun, and Cunni Zheng provided technical support. Quan Liu was involved in manuscript revision and editing, funding acquisition, and project management. All authors reviewed the manuscript.

## Ethics Statement

The animal study was approved by the Laboratory Animal Centre of Southern University of Science and Technology. All experimental procedures were performed in compliance with the institutional guidelines for the care and use of laboratory animals, as well as relevant national regulations.

## Conflicts of Interest

The authors declare no conflicts of interest.

## Supporting information


Supporting Information S1

## Data Availability

The data that support the findings of this study are available from the corresponding author upon reasonable request.
