# Supplementary material for: ST2+ Erythroid Progenitors Suppress Allergic Asthma by Scavenging IL‐33 in Young Mice
Source: Clin Transl Allergy. 2025 Oct 17;15(10):e70111. doi: 10.1002/clt2.70111 (PMC12534176; doi:10.1002/clt2.70111)
Supplement: Supplementary file 1 — Supporting Information S1 [file CLT2-15-e70111-s001.docx]

**Supplementary material**

**ST2^+^ erythroid progenitors suppress allergic asthma by scavenging IL-33 in young mice**

Chang Li^1^, Jie Liu^1,2^, Xiaoshi Li^1^, Wenlong Chen^1^, Ying Zhang^1^, Heng Sun^1,3^, Cunni Zheng^1^, and Quan Liu^1*^

^1^Department of Biochemistry, School of Medicine, Southern University of Science and Technology, Shenzhen, Guangdong Province 518116, China;

^2^ Flow Cytometry Core Facility, Shenzhen Medical Academy of Research and Translation, Shenzhen, Guangdong Province 518107, China;

^3^Department of Oncology, National Cancer Center/National Clinical Research Center for Cancer/Cancer Hospital & Shenzhen Hospital, Chinese Academy of Medical Sciences and Peking Union Medical College, Shenzhen, Guangdong Province 518116, China.

***Corresponding Author:**

Quan Liu, M.D., Ph.D.

Address:Department of Biochemistry, School of Medicine, Southern University of Science and Technology

1088 Xueyuan Avenue, Shenzhen, Guangdong Province 518055, China

Phone: +86-755-88018055

Email: [liuq3@sustech.edu.cn](mailto:liuq3@sustech.edu.cn)

**Supplementary figures:**


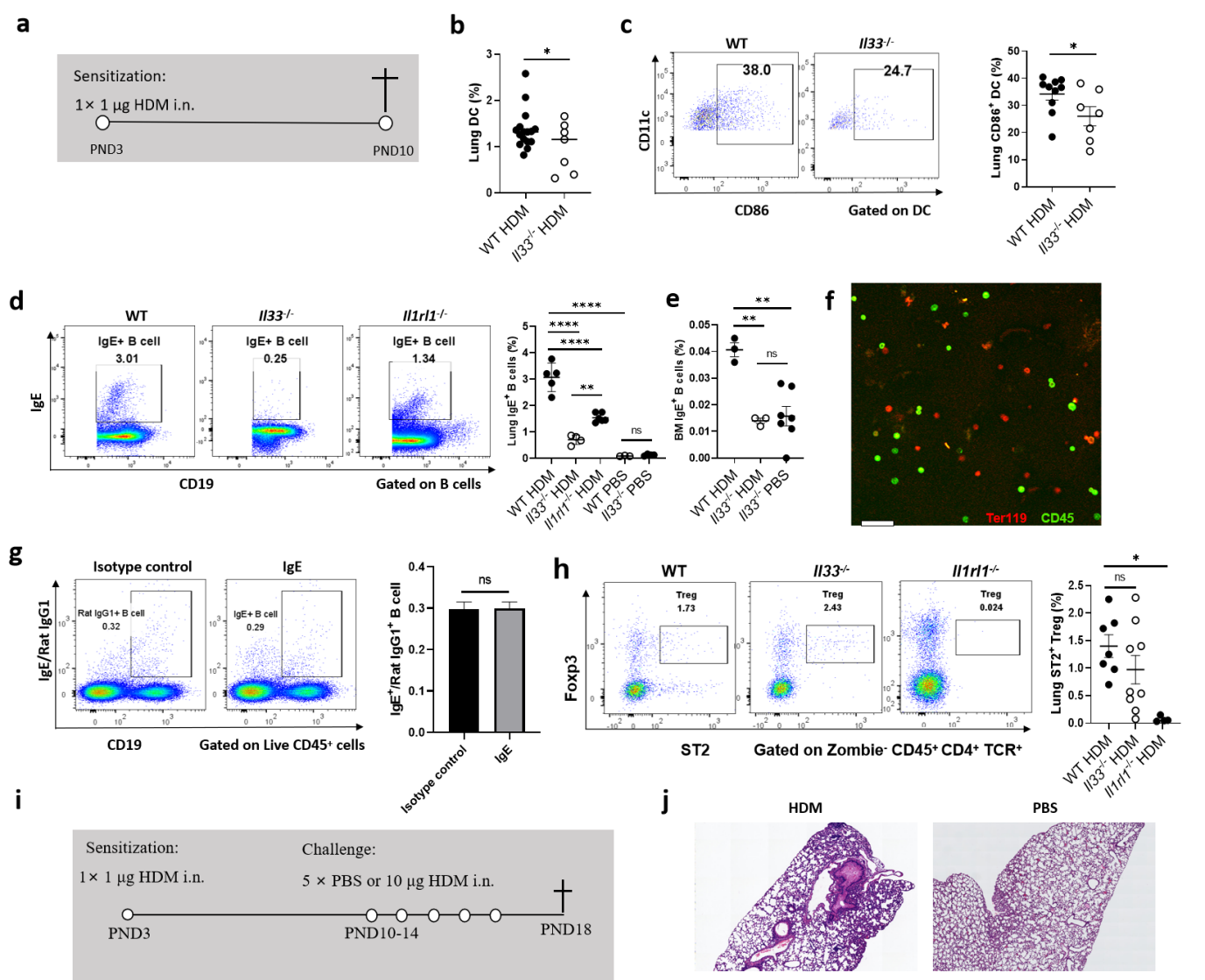


**Extended Data Figure 1 |Differences in immune cell populations in neonatal mice after HDM-Induced sensitization or asthma.**
**(a)** Schematic of the HDM-induced sensitization model in young mice. **(b)** Proportion of lung dendritic cells (DCs) (Zombie⁻ CD45^+^ CD11c^+^ IA/IE^+^). **(c)** Proportion of CD86^+^ lung DCs (Zombie⁻ CD45^+^ CD11c^+^ IA/IE^+^ CD86^+^). **(d)** Proportion of IgE^+^ B cells in lungs (Zombie⁻ CD45^+^ CD19^+^ IgE^+^) following HDM or PBS sensitization. **(e)** Proportion of IgE^+^ B cells in BM (Zombie⁻ CD45^+^ CD19^+^ IgE^+^). (f) Confocal imaging of the sorted splenic CD45^+^ Ter119^+^ cells.  **(g)** IgE^+^ B cell proportion in MLN (Zombie⁻ CD45^+^ CD19^+^ IgE^+^/ Rat IgG1^+^), n=3. **(h)** Proportion of Treg in lungs (Zombie⁻ CD45^+^ CD4^+^ TCRβ^+^) following HDM sensitization.**(i)** Schematic of the HDM-induced asthma model in young mice. (j) Lung H&E staining of HDM-induced allergic asthma mice.Data are mean ± SEM; ns, *p < 0.05, **p < 0.01, ***p < 0.001, ****p < 0.0001.


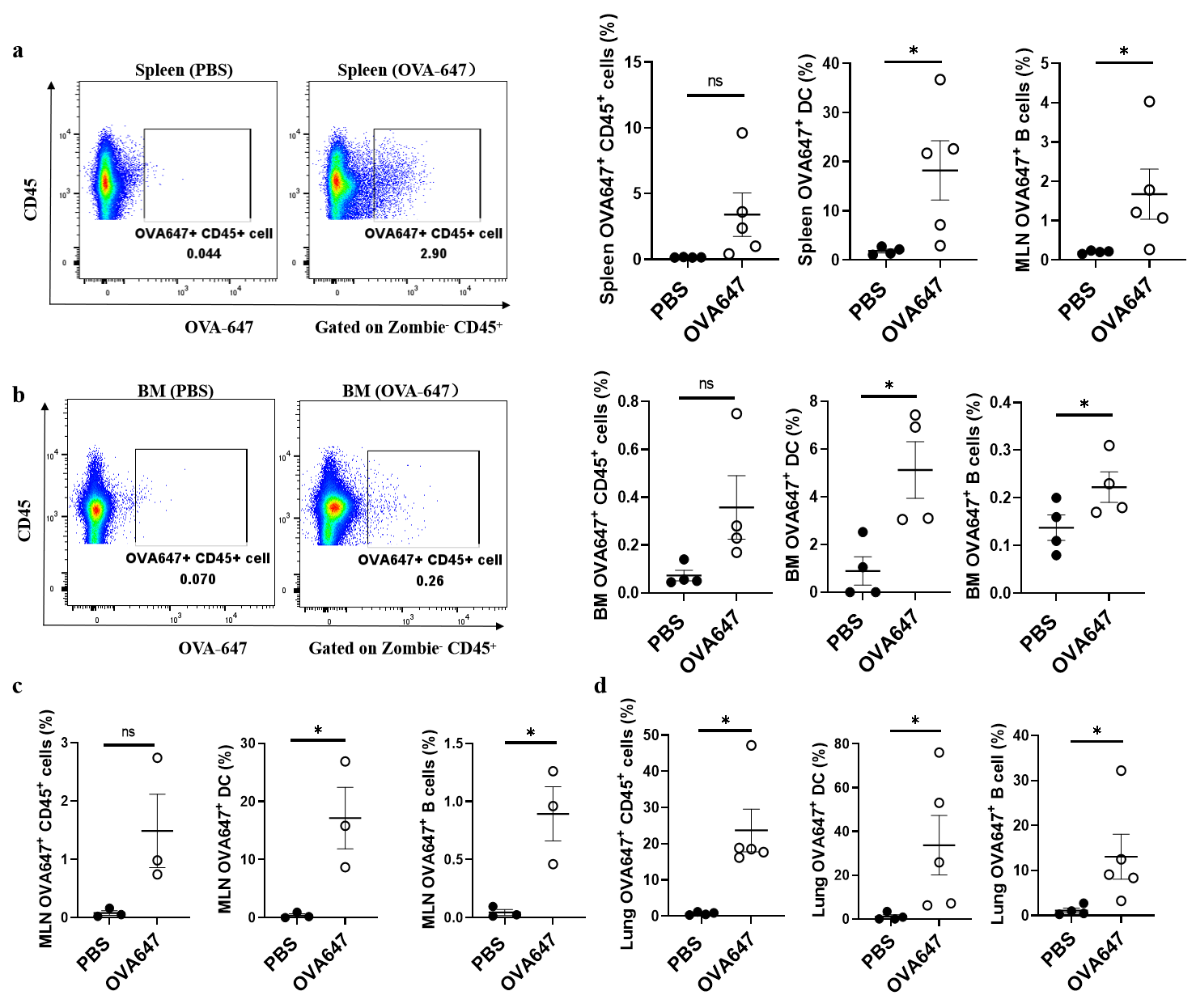


**Extended Data Figure 2 |** **OVA647 distribution in young mouse tissues 24h post-intranasal administration (100 µg)**

(a-d) Proportion of OVA-647^+^ CD45^+^ cells (Zombie⁻ CD45^+^ OVA-647^+^), dendritic cells (DCs; Zombie⁻ CD45^+^ CD11c^+^ IA/IE^+^), and B cells (Zombie⁻ CD45^+^ CD19^+^) in: (a) spleen, (b) BM, (c) MLN, (d) lung. and. Data are mean ± SEM; ns, *p < 0.05.


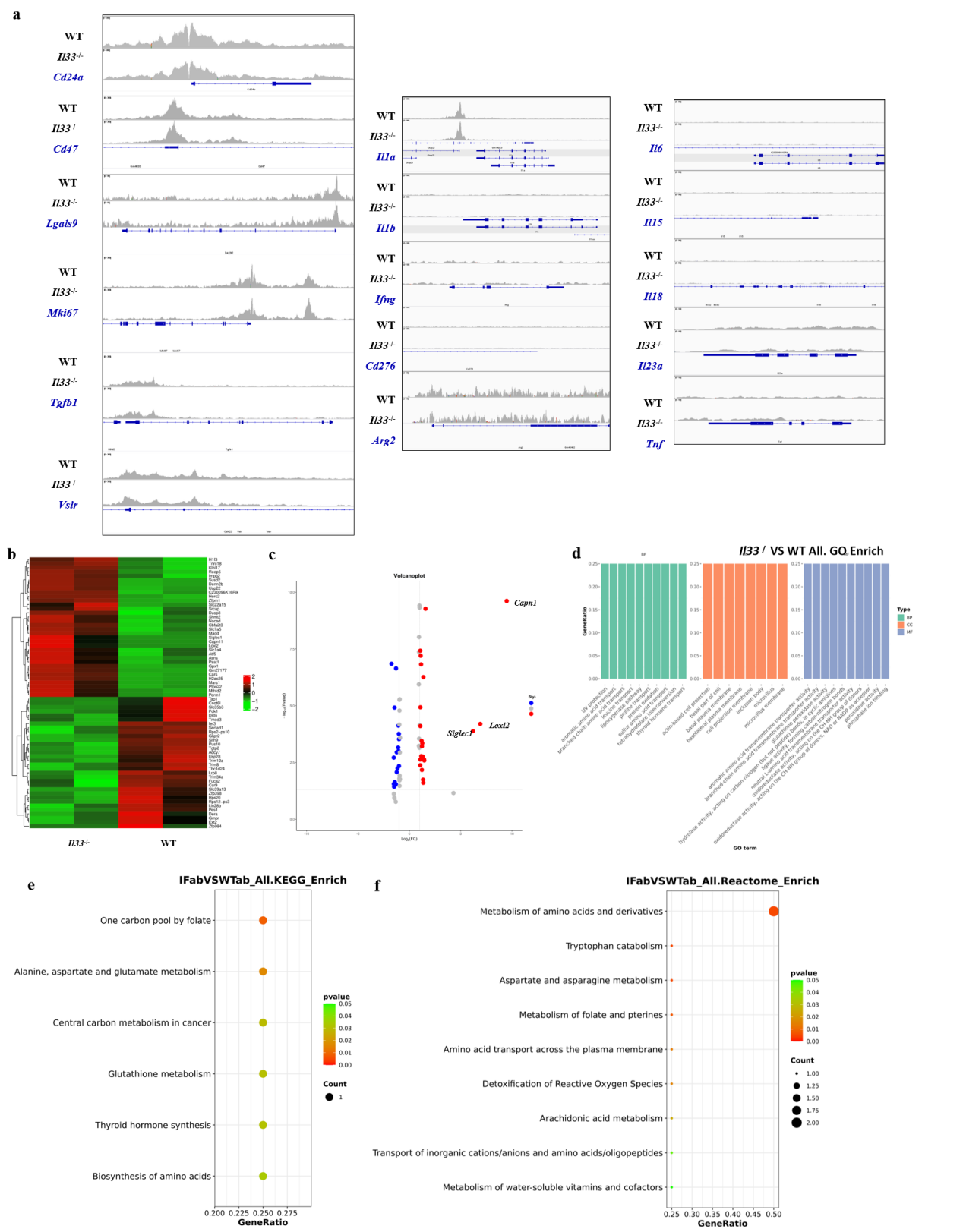


**Extended Data Figure 3 | Epigenomic and transcriptomic profiling of splenic ST2^+^ EPs in WT versus *Il33*^⁻/⁻^ young mice.**

**(a) Chromatin accessibility landscape of immune-related genes in ST2^+^ EPs from WT and *Il33*^⁻/⁻^ mice (ATAC-Seq). (b) Heatmap analysis of differentially expressed genes in splenic ST2^+^ EPs from PND7 WT and *Il33*^⁻/⁻^ mice (RNA-Seq). (c) Volcano plot analysis of gene expression differences between WT and *Il33*^⁻/⁻^ ST2^+^ EPs (RNA-Seq). (d) Gene Ontology (GO) enrichment analysis of differentially expressed genes. (e) KEGG pathway enrichment analysis of transcriptomic data. (f) Reactome pathway analysis of gene expression profiles.**


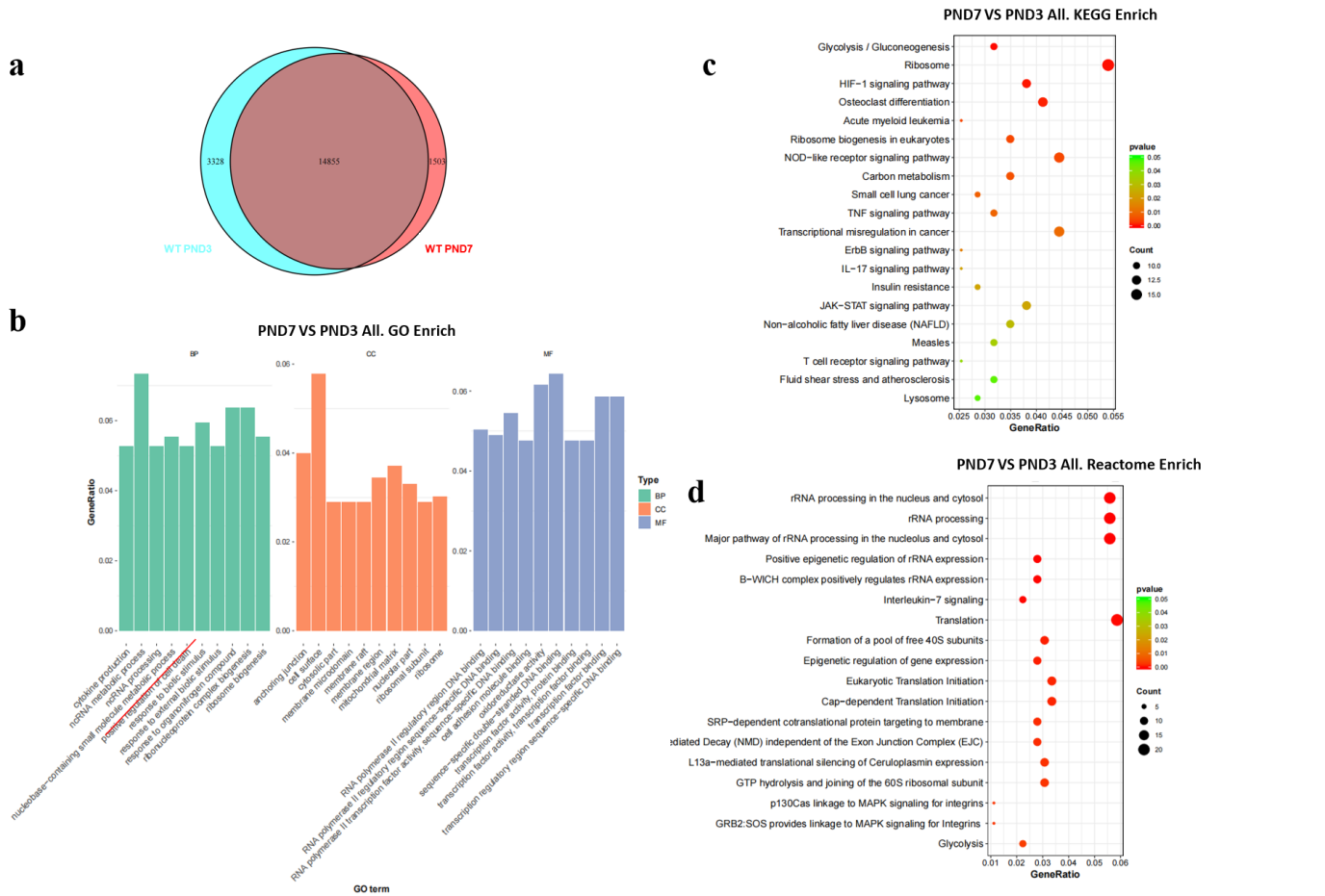


**Extended Data Figure 4 | Transcriptomic study of splenic ST2^+^ EPs in WT mice at PND 3 and PND 7.**

**(a) Venn diagram showing genes of splenic ST2^+^ EPs in WT mice at PND 3 and PND 7 based on RNA-Seq data. (b) Gene Ontology (GO) enrichment analysis of genes of splenic ST2^+^ EPs in WT mice at PND 3 and PND 7 based on RNA-Seq data. (c) KEGG pathway enrichment analysis of genes of splenic ST2^+^ EPs in WT mice at PND 3 and PND 7 based on RNA-Seq data. (d) Reactome pathway analysis of genes of splenic ST2^+^ EPs in WT mice at PND 3 and PND 7 based on RNA-Seq data.**

**Supplementary tables:**

Table S1　Antibody Information

| Antibody name | Clone | Resource | Cat number | RRID number |
| --- | --- | --- | --- | --- |
| BUV395 Rat Anti-Mouse Ly-6G Antibody | 1A8 (RUO) | BD Horizon™ | 563978 | AB_2716852 |
| BUV395 Rat Anti-Mouse CD90.2 Antibody | 53-2.1 (RUO | BD Horizon™ | 565257 | AB_2739136 |
| BUV395 Rat Anti-Mouse CD8a Antibody | 53-6.7 (RUO | BD Horizon™ | 563786 | AB_2732919 |
| BUV395 Rat Anti-Mouse Gr-1 | RB6-8C5 (RUO) | BD Horizon™ | 563849 | AB_2738450 |
| BUV395 Hamster Anti-Mouse CD11c | HL3 (RUO) | BD Horizon™ | 564080 | AB_2738580 |
| Antibody name | Clone | Resource | Cat number | RRID number |
| BUV737 Hamster Anti-Mouse TCR β Chain | H57-597 (RUO) | BD Horizon™ | 612821 |  |
| BUV737 Rat Anti-Mouse TER-119/Erythroid Cells | TER-119 (RUO) | BD Horizon™ | 741736 | AB_2871106 |
| BUV737 Rat Anti-CD11b Antibody | M1/70 (RUO) | BD Horizon™ | 612800 | AB_2738811 |
| T-bet Monoclonal Antibody，eFluor™ 450 | eBio4B10 (4B10) | eBioscience™ | 48-5825-82 | AB_2784727 |
| Pacific Blue™ anti-mouse I-A/I-E Antibody | [M5/114.15.2](https://www.biolegend.com/en-gb/search-results?Clone=M5/114.15.2) | BioLegend | 107620 | AB_493527 |
| Pacific Blue™ anti-mouse CD4 Antibody | [RM4-5](https://www.biolegend.com/en-gb/search-results?Clone=RM4-5) | BioLegend | 100531 | AB_493374 |
| Pacific Blue™ anti-mouse TCR β chain Antibody | [H57-597](https://www.biolegend.com/en-gb/search-results?Clone=H57-597) | BioLegend | 109226 | AB_1027649 |
| Pacific Blue™ anti-mouse CD90.2 (Thy1.2) Antibody | [53-2.1](https://www.biolegend.com/en-gb/search-results?Clone=53-2.1) | BioLegend | 140306 | AB_10641693 |
| Brilliant Violet 421™ anti-mouse CD135 Antibody | A2F10 | BioLegend | 135313 | AB_2562338 |
| Brilliant Violet 421™ anti-mouse CD34 Antibody | [SA376A4](https://www.biolegend.com/en-gb/search-results?Clone=SA376A4) | BioLegend | 152208 | AB_2650765 |
| Brilliant Violet 421™ anti-mouse F4/80 | BM8 | BioLegend | 123131 | AB_10901171 |
| Pacific Blue™ anti-mouse CD8a Antibody | [53-6.7](https://www.biolegend.com/en-gb/search-results?Clone=53-6.7) | BioLegend | 100725 | AB_493425 |
| BV421 Hamster Anti-Mouse CD11c Antibody | HL3 | BD Horizon™ | 562782 | AB_2737789 |
| BV421 Rat Anti-Mouse IL-33R (ST2) Antibody | U29-93 | BD Horizon™ | 566309 | AB_2744489 |
| Brilliant Violet 510™ anti-mouse CD4 Antibody | [RM4-5](https://www.biolegend.com/en-gb/search-results?Clone=RM4-5) | BioLegend | 100553 | AB_2561388 |
| Antibody name | Clone | Resource | Cat number | RRID number |
| Brilliant Violet 510™ anti-mouse CD45 Antibody | [30-F11](https://www.biolegend.com/en-gb/search-results?Clone=30-F11) | BioLegend | 103137 | AB_2561392 |
| BV605 Rat Anti-Mouse Siglec-FAntibody | E50-2440 | BD Horizon™ | 740388 | AB_2740118 |
| Brilliant Violet 605™ anti-mouse CD4Antibody | RM4-5 | BioLegend | 100547 | AB_11125962 |
| Brilliant Violet 605™ anti-mouse F4/80 Antibody | BM8 | BioLegend | 123133 | AB_2562305 |
| BV605 Rat Anti-CD11b | M1/70 | BD Horizon™ | 563015 | AB_2737951 |
| FITC anti-mouse TCR β chain Antibody | [H57-597](https://www.biolegend.com/en-gb/search-results?Clone=H57-597) | BioLegend | 109206 | AB_313428 |
| FITC anti-mouse/human CD11b Antibody | [M1/70](https://www.biolegend.com/en-gb/search-results?Clone=M1/70) | BioLegend | 101206 | AB_312788 |
| FITC anti-mouse CD185 (CXCR5) Antibody | [L138D7](https://www.biolegend.com/en-gb/search-results?Clone=L138D7) | BioLegend | 145519 | AB_2562865 |
| Alexa Fluor® 488 anti-mouse CD8a Antibody | [53-6.7](https://www.biolegend.com/en-gb/search-results?Clone=53-6.7) | BioLegend | 100723 | AB_389304 |
| Alexa Fluor® 488 anti-mouse CD45 Antibody | 30-F11 | BioLegend | 103122 | AB_493531 |
| Alexa Fluor® 488 anti-mouse CD4 Antibody | [RM4-5](https://www.biolegend.com/en-gb/search-results?Clone=RM4-5) | BioLegend | 100529 | AB_493373 |
| Alexa Fluor® 488 anti-mouse TER-119/Erythroid Cells Antibody | [TER-119](https://www.biolegend.com/en-gb/search-results?Clone=TER-119) | BioLegend | 116215 | AB_493402 |
| T1/ST2 (IL-33R) Mouse，Monoclonal Antibody，FITCAntibody | DJ8 | MD Bioproducts | 101001F |  |
| PerCP/Cyanine5.5 anti-mouse CD11c Antibody | [N418](https://www.biolegend.com/en-gb/search-results?Clone=N418) | BioLegend | 117328 | AB_2129641 |
| Antibody name | Clone | Resource | Cat number | RRID number |
| PerCP/Cyanine5.5 anti-mouse CD19 | [1D3/CD19](https://www.biolegend.com/en-gb/search-results?Clone=1D3/CD19) | BioLegend | 152406 | AB_2629814 |
| PerCP/Cyanine5.5 anti-mouse CD8a | [53-6.7](https://www.biolegend.com/en-gb/search-results?Clone=53-6.7) | BioLegend | 100734 | AB_2075239 |
| PerCP/Cyanine5.5 anti-mouse I-A/I-E | [M5/114.15.2](https://www.biolegend.com/en-gb/search-results?Clone=M5/114.15.2) | BioLegend | 107626 | AB_2191071 |
| PerCP/Cyanine5.5 anti-mouse CD4 | [RM4-5](https://www.biolegend.com/en-gb/search-results?Clone=RM4-5) | BioLegend | 100540 | AB_893326 |
| PerCP/Cyanine5.5 anti-mouse/human CD11b Antibody | M1/70 | BioLegend | 101228 | AB_893232 |
| PerCP-Cy™5.5 Rat Anti-Mouse CD24 | M1/69 | BD Pharmingen™ | 562360 | AB_11151895 |
| PE anti-mouse IgE | [RME-1](https://www.biolegend.com/en-gb/search-results?Clone=RME-1) | BioLegend | 406908 | AB_493290 |
| PE Rat IgG1, κ Isotype Ctrl | RTK2071 | BioLegend | 400407 | AB_326513 |
| PE anti-mouse CD4 | [RM4-5](https://www.biolegend.com/en-gb/search-results?Clone=RM4-5) | BioLegend | 100512 | AB_312714 |
| PE anti-mouse/human CD11b Antibody | [M1/70](https://www.biolegend.com/en-gb/search-results?Clone=M1/70) | BioLegend | 101208 | AB_312790 |
| T1/ST2 (IL-33 R) Mouse，Monoclonal Antibody，PE | DJ8 | MD Bioproducts | 101001PE |  |
| PE anti-mouse CD117 (c-Kit) | 2B8 | BioLegend | 105808 | AB_313216 |
| PE anti-mouse NK-1.1 Antibody | PK136 | BioLegend | 108708 | AB_313394 |
| PE/Cyanine7 anti-mouse CD86 | GL-1 | BioLegend | 105014 | AB_439782 |
| FOXP3 Monoclonal Antibody (FJK-16s)，PerCP-Cyanine5.5 | FJK-16s | eBioscience™ | 45-5773-82 | AB_914351 |
| PE-Cy™7 Hamster Anti-Mouse CD3e Antibody | 145-2C11 | BioLegend | 552774 | AB_394460 |
| Purified anti-mouse CD16/32 Antibody | 93 | BioLegend | 101302 | AB_312800 |
| PE/Cyanine7 anti-mouse CD127 (IL-7Rα) Antibody | A7R34 | BioLegend | 135014 | AB_1937265 |
| Antibody name | Clone | Resource | Cat number | RRID number |
| PE/Cyanine7 anti-mouse F4/80 Antibody | BM8 | BioLegend | 123114 | AB_893478 |
| PE/Cyanine7 anti-mouse TCR β chain Antibody | H57-597 | BioLegend | 109222 | AB_893627 |
| PE-Cyanine7 CD279 (PD-1) Monoclonal Antibody (J43) | J43 | eBioscience™ | 25-9985-82 | AB_10853805 |
| PE/Cyanine7 anti-mouse CD71 Antibody | RI7217 | BioLegend | 113812 | AB_2203383 |
| PE/Dazzle™ 594 anti-mouse/human KLRG1 (MAFA) Antibody | 2F1/KLRG1 | BioLegend | 138424 | AB_2564050 |
| PE/Dazzle™ 594 anti-mouse CD11c  Antibody | N418 | BioLegend | 117348 | AB_2563654 |
| PE/Cyanine7 anti-mouse CD117 (c-Kit) Antibody | 2B8 | BioLegend | 105814 | AB_313222 |
| APC FcεR1 alpha Monoclonal Antibody | MAR-1 | [eBioscience™](https://www.thermofisher.cn/antibody/product/FceR1-alpha-Antibody-clone-MAR-1-Monoclonal/17-5898-82) | 17-5898-82 | AB_10718824 |
| APC anti-mouse CD11c Antibody | N418 | BioLegend | 117310 | AB_313778 |
| APC anti-mouse CD71 Antibody | RI7217 | BioLegend | 113820 | AB_2728134 |
| APC anti-mouse CD185 (CXCR5) Antibody | L138D7 | BioLegend | 145506 | AB_2561969 |
| APC anti-mouse TER-119/Erythroid Cells Antibody | TER-119 | BioLegend | 116212 | AB_313712 |
| Alexa Fluor® 647 anti-mouse Ly-6G | 1A8 | BioLegend | 127610 | AB_1134159 |
| Alexa Fluor® 647 Mouse anti-GATA3 | L50-823 | BD Pharmingen™ | 560078 | AB_1645317 |
| Alexa Fluor® 647 Rat anti-Mouse CD34 | RAM34 | BD Pharmingen™ | 560230 | AB_1645199 |
| Antibody name | Clone | Resource | Cat number | RRID number |
| Alexa Fluor® 647 anti-mouse F4/80 | BM8 | BioLegend | 123122 | AB_893480 |
| Alexa Fluor® 647 anti-mouse TCR β chain Antibody | H57-597 | BioLegend | 109218 | AB_493346 |
| Alexa Fluor® 647 anti-mouse CD127 (IL-7Rα) Antibody | A7R34 | BioLegend | 135020 | AB_1937210 |
| Super Bright™ 600 CD150 Monoclonal | mShad150 | eBioscience™ | 63-1502-82 | AB_2734884 |
| DyLight™ 649 Donkey anti-rabbit IgG Antibody | Poly4064 | BioLegend | 406406 | AB_1575135 |
| APC/Cyanine7 anti-mouse CD19 Antibody | 6D5 | BioLegend | 115530 | AB_830706 |
| APC/Cyanine7 anti-mouse CD90.2 (Thy1.2) Antibody | 53-2.1 | BioLegend | 140332 | AB_2894662 |
| APC/Fire™ 750 anti-mouse NK-1.1 Antibody | PK136 | BioLegend | 108752 | AB_2629764 |
| APC/Fire™ 750 anti-mouse CD11c | N418 | BioLegend | 117352 | AB_2572124 |
| APC/Fire™ 750 anti-mouse TCR β chain Antibody | H57-597 | BioLegend | 109246 | AB_2629696 |
| APC/Fire™ 750 anti-mouse CD8a | 53-6.7 | BioLegend | 100766 | AB_2572113 |
| APC/Fire™ 750 anti-mouse/human CD11b Antibody | M1/70 | BioLegend | 101262 | AB_2572121 |
| APC/Fire™ 750 anti-mouse Ly-6G/Ly-6C (Gr-1) Antibody | [RB6-8C5](https://www.biolegend.com/de-de/search-results?Clone=RB6-8C5) | BioLegend | 108456 | AB_2616737 |
| APC/Fire™ 750 anti-mouse/human CD45R/B220 Antibody | [RA3-6B2](https://www.biolegend.com/de-de/search-results?Clone=RA3-6B2) | BioLegend | 103260 | AB_2572108 |
| APC-eFluor™ 780 FcεR1 alpha Monoclonal Antibody | MAR-1 | eBioscience™ | 47-5898-82 | AB_2573990 |
| Antibody name | Clone | Resource | Cat number | RRID number |
| Alexa Fluor® 647 Conjugate Phospho-NF-κB p65 (Ser536) Rabbit mAb | 93H1 | Cell signaling | 4887s |  |
| Alexa Fluor® 647 Conjugate Phospho-p38 MAPK (Thr180/Tyr182) Mouse mAb | 28B10 | Cell signaling | 4552S |  |
| Alexa Fluor® 647 Conjugate Phospho-SAPK/JNK (Thr183/Tyr185) Mouse mAb | G9 | Cell signaling | 9257s |  |
| Alexa Fluor® 647 Conjugate Phospho-p44/42 MAPK (Erk1/2) (Thr202/Tyr204) Rabbit mAb | 197G2 | Cell signaling | 13148s |  |
| eFluor™ 660 Gata-3 Monoclonal Antibody | TWAJ | eBioscience™ | 50-9966-42 | AB_10596663 |
| APC anti-mouse CD4 Antibody | RM4-5 | BioLegend | 100516 | AB_312718 |
| DAPI |  | R＆D | 2507021 |  |
| Mouse IL-33 Antibody Polyclonal Goat IgG |  | R＆D | AF3626 |  |
| Purified anti-GAPDH Antibody | W17079A | BioLegend | 607902 | AB_2734503 |
| HRP-conjugated Donkey Anti-Goat  IgG(H+L) |  | Proteintech | SA00001-3 | AB_2890882 |
| Zombie NIR™ Fixable Viability Kit |  | BioLegend | 423106 |  |
| Donkey anti-Goat Alexa Fluor™ Plus 594 IgG (H+L) Highly Cross-Adsorbed Secondary Antibody |  | Invitrogen | A32758 | AB_2762828 |

Table S2　 Materials Information

| Name | Resource | Cat number |
| --- | --- | --- |
| Foxp3 / Transcription Factor Staining Buffer Set | eBioscience™ | 2507021 |
| Brilliant Stain Buffer Plus | BD Horizon™ | 566385 |
| Anti-fluorescence quenching PVP mounting medium | Beyotime | P0123 |
| UltraSignal Ultra-Sensitive ECL Chemiluminescent Substrate | 4A Biotech | 4AW011 |
| Mouse IL-33 Uncoated ELISA | Thermo | 88-7333 |
| InVivoMAb anti-mouse CD71 (TfR1) | Bio X cell | BE0329 |
| InVivoMAb ratigG2a isotype control | Bio X cell | BE0089 |
| HDM House Dust Mite | GREER | XPB91D3A25 |
| Trypsin-EDTA (0.25%)，phenol red | Gibco™ | 25200072 |
| OVA，Alexa Fluor 647 | invitrogen | O34784 |
| 2-methylbutane | Macklin | M822968-1L |
| BASIC DMEM，High Glucose，Pyruvate | Gibco™ | C11995500BT |
| RPMI Medium 1640 basic (1X) | Gibco™ | C11875500BT |
| Sodium heparin | Solarbio | H8060-5g |
| FBS | Gibco™ | 10099141C |
| Sucrose | Sigma | V900116 |
| Matrigel | Corning | 354234 |
| O.C.T. Compound | Servicebio | G6059-110ML |
| MojoSort™ Mouse CD4 T Cell Isolation Kit | BioLegend | 480033 |
| Stemspan SFEM medium | Stemcell | 09650 |
| PBS，1X，pH 7.4 | Gibco™ | C10010500BT |
| Cell Stimulation Cocktail (plus protein transport inhibitors） (500× | Thermo Scientific | 00-4975-03 |
| Name | Resource | Cat number |
| Recombinant mouse IL-33(carrier-free） | BioLegend | 580508 |
| TRIzol | Thermo | 5596026CN |
